# Assessment of glucagon receptor occupancy by Positron Emission Tomography in non-human primates

**DOI:** 10.1038/s41598-019-51530-0

**Published:** 2019-10-18

**Authors:** Olof Eriksson, Irina Velikyan, Torsten Haack, Martin Bossart, Andreas Evers, Iina Laitinen, Philip J. Larsen, Oliver Plettenburg, Akihiro Takano, Christer Halldin, Gunnar Antoni, Lars Johansson, Stefan Pierrou, Michael Wagner

**Affiliations:** 1 Antaros Medical AB, Mölndal, Sweden; 2 Science For Life Laboratory, Department of Medicinal Chemistry, Uppsala University, Uppsala, Sweden; 3 PET Centre, Centre for Medical Imaging, Uppsala University Hospital, Uppsala, Sweden; 4 Department of Medicinal Chemistry, Uppsala University, Uppsala, Sweden; 5 Sanofi-Aventis, Frankfurt, Germany; 6 Bayer Pharmaceuticals, Wuppertal, Germany; 7 Institute of Medicinal Chemistry, Helmholtz Zentrum München, German Research Center for Environmental Health (GmbH), Neuherberg, Germany; 8 Institute of Organic Chemistry, Leibniz Universität Hannover, Hannover, Germany; 9 Department of Clinical Neuroscience, Center for Psychiatry Research, Karolinska Institutet and Stockholm County Council, Stockholm, Sweden; 10 Lee Kong Chian School of Medicine, Nanyang Technological University, Singapore, Singapore

**Keywords:** Predictive markers, Predictive markers

## Abstract

The glucagon receptor (GCGR) is an emerging target in anti-diabetic therapy. Reliable biomarkers for *in vivo* activity on the GCGR, in the setting of dual glucagon-like peptide 1/glucagon (GLP-1/GCG) receptor agonism, are currently unavailable. Here, we investigated [^68^Ga]Ga-DO3A-S01-GCG as a biomarker for GCGR occupancy in liver, the tissue with highest GCGR expression, in non-human primates (NHP) by PET. [^68^Ga]Ga-DO3A-S01-GCG was evaluated by dynamic PET in NHPs by a dose escalation study design, where up to 67 µg/kg DO3A-S01-GCG peptide mass was co-injected. The test-retest reproducibility of [^68^Ga]Ga-DO3A-S01-GCG binding in liver was evaluated. Furthermore, we investigated the effect of pre-treatment with acylated glucagon agonist 1-GCG on [^68^Ga]Ga-DO3A-S01-GCG binding in liver. [^68^Ga]Ga-DO3A-S01-GCG bound to liver *in vivo* in a dose-dependent manner. Negligible peptide mass effect was observed for DO3A-S01-GCG doses <0.2 µg/kg. *In vivo* K_d_ for [^68^Ga]Ga-DO3A-S01-GCG corresponded to 0.7 µg/kg, which indicates high potency. The test-retest reproducibility for [^68^Ga]Ga-DO3A-S01-GCG binding in liver was 5.7 ± 7.9%. Pre-treatment with 1-GCG, an acylated glucagon agonist, resulted in a GCGR occupancy of 61.5 ± 9.1% in liver. Predicted human radiation dosimetry would allow for repeated annual [^68^Ga]Ga-DO3A-S01-GCG PET examinations. In summary, PET radioligand [^68^Ga]Ga-DO3A-S01-GCG is a quantitative biomarker of *in vivo* GCGR occupancy.

## Introduction

The glucagon receptor (GCGR) is an emerging target in anti-diabetic therapy. Dual Glucagon-Like Peptide-1 (GLP-1)/glucagon (GCG) receptor agonists have shown promising therpeutic effects, including weight loss and glycaemic control in preclinical and early clinical settings^1–3^. The lack of quantitative biomarkers for monitoring *in vivo* occupancy at the GCGR, as part of the overlapping dual pharmacology of GLP-1/GCG receptor agonists, makes drug candidate selection and optimisation difficult. Direct measurement of occupancy of each receptor as a function of *in vivo* exposure could help to unravel the pharmacological basis of the different physiological effects of GLP-1/GCG receptor agonists. The Positron Emission Tomography (PET) radioligand [^68^Ga]Ga-DO3A-VS-Cys40-Exendin-4 was previously developed for quantification of GLP-1R occupancy in the pancreas^4^.

**Figure 1:**
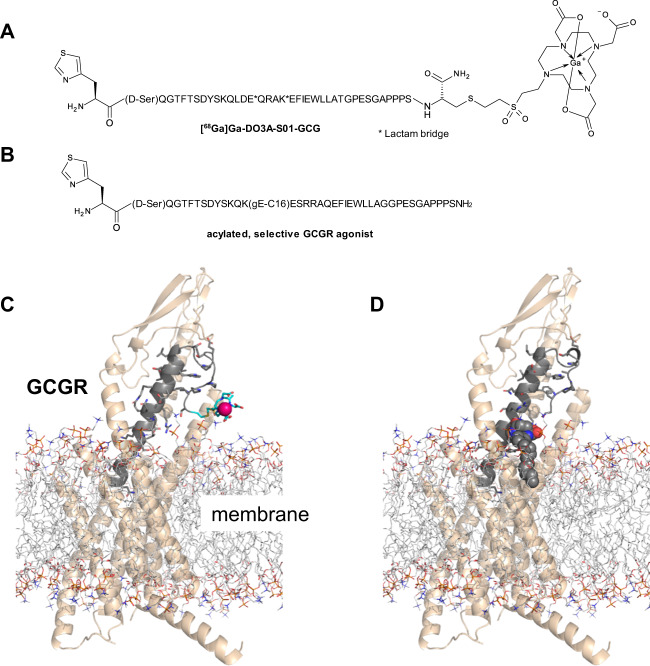
Structures and GCGR binding model of [^68^Ga]Ga-DO3A-S01-GCG (**A**,**C**) and the acylated selective GCGR agonist 1-GCG (**B**,**D**). The binding model is based on the full-length structure of the GCGR (pdb code: 5yqz). Structural receptor-peptide binding models and images were generated using maestro and PyMOL (Schrödinger, LLC, New York, NY).

Recently, we presented a series of first-in-class GCGR PET radioligands, based on rationally designed peptide sequences, with high affinity and specificity towards the GCGR^5,6^. The analogues labelled with Gallium-68 maintained suitable affinity and selectivity for the GCGR and demonstrated negligible off-target binding on the GLP-1 receptor. One of the analogues, in particular, [^68^Ga]Ga-DO3A-S01-GCG (Fig. 1A,C), exhibited strong specific *in vivo* binding in rat liver, the relevant tissue for GCGR directed therapy.

In this study, we further evaluated [^68^Ga]Ga-DO3A-S01-GCG in non-human primate (NHP) as a first-in-class, quantitative biomarker of *in vivo* GCGR occupancy.

## Materials and Methods

### Radiochemistry

Gallium-68 radiolabeling of DO3A-S01-GCG (Fig. 1A) was performed as described previously^5^.

### Non-human primate handling, housing and anaesthesia

PET/CT imaging was performed in NHPs (cynomolgus monkeys, n = 12, 11 female and 1 male, weight 5.1 ± 1.1, range 3.4–6.7 kg), which were housed in the Astrid Fagraeus Laboratory (AFL) at the Karolinska Institutet, Solna, Sweden. They were transported to the PET facility at the morning of each experiment. Two different PET imaging facilities were used in this study: Uppsala University Hospital (UUH) and Karolinska Institutet PET centre (KI).

Anaesthesia was induced by intramuscular injection of ketamine hydrochloride (10 mg/kg) at the housing facility. Two venous catheters were applied after induction of anaesthesia; one for PET radiopharmaceutical administration and one for blood sampling. Anaesthesia was maintained by the administration of a mixture of 1.5–2.0% isoflurane (KI) or 1–4% sevoflurane (UUH), oxygen and medical air after endotracheal intubation at each PET centre. Body temperature was maintained throughout the examination by a Bair Hugger (Arizant Healthcare, MN). Body temperature, ECG, heart rate, respiratory rate, oxygen saturation, blood pressure and B-glucose were monitored throughout the experiments. Fluid balance was maintained by a continuous infusion of saline or Ringer’s Acetate, supplemented with glucose if needed. The procedures involving NHPs at both sites were approved by the Animal Ethics Committee of the Swedish Animal Welfare Agency and carried out in accordance with the relevant national and institutional guidelines (UU: “Uppsala university guidelines on animal experimentation”, UFV 2007/724, KI: “Guidelines for planning, conducting and documenting experimental research”, Dnr 4820/06-600).

### PET procedures: dose escalation studies

Dose escalation studies were performed in seven NHPs (n = 7, weight 5.0 ± 1.1, range 3.4–6.7 kg) with up to three sequential PET examinations performed in each NHP for each experimental day. The examinations were performed 2–3 hours apart to allow for radioactivity excretion and radionuclide decay (t_½_ = 68 minutes). Each subsequent administration contained gradually increasing amounts of co-injected DO3A-S01-GCG peptide, thus, respectively decreasing the [^68^Ga]Ga-DO3A-S01-GCG tracer molar activity (previously referred to as specific radioactivity) value (MBq/nmol) (Table 1). For the high competing doses, unlabelled DO3A-S01-GCG was added to the [^68^Ga]Ga-DO3A-S01-GCG injection solution in order to reach the desired peptide mass.

**Table 1:** Dosing of [^68^Ga]Ga-DO3A-S01-GCG and corresponding co-injection of DO3A-S01-GCG peptide mass for each NHP experimental day.

ID		NHP 1	NHP 2	NHP 3	NHP 4	NHP 5	NHP 6	NHP 7
	Weight (kg)	5.4	6.0	5.4	6.7	4.4	3.4	4.1
Scan 1	µg/kg	0.2	0.09	0.08	0.05	0.045	0.051	0.044
	MBq/kg	1.0	1.3	0.6	0.4	0.7	0.8	0.7
Scan 2	µg/kg	13	1.0	2.7	0.47	—	—	—
	MBq/kg	3.2	2.6	3.3	0.7	—	—	—
Scan 3	µg/kg	27	6	67	61	—	—	—
	MBq/kg	7.9	9.9	10.1	8	—	—	—

Each animal was positioned to include the abdomen in the Field of View (FOV).

At the UUH site, the examination was conducted using a Discovery IQ PET/CT scanner (15 cm FOV, approximately 5 mm resolution, GE Healthcare, Milwaukee, MI, USA). Attenuation correction was acquired by a 140 kV, Auto mA 10–80 mA CT examination. Dynamic PET measurements were acquired over 90 minutes in list mode.

At the KI facility, stand-alone PET measurements were conducted using a High Resolution Research Tomograph (HRRT) (approximately 3 mm resolution, Siemens Molecular Imaging). A transmission scan of 6 min using a single ^137^Cs source was performed for attenuation correction of PET images. Dynamic PET measurements were acquired over 93 minutes in list mode.

Venous blood samples for radioactivity measurement in whole blood and plasma were taken 5, 30 and 60 min after [^68^Ga]Ga-DO3A-S01-GCG radioactivity administration, to calculate the plasma-to-whole blood concentration ratios.

For NHP 5–7, venous blood samples were taken at 5, 30 and 60 min for assessment of *in vivo* metabolic stability of [^68^Ga]Ga-DO3A-S01-GCG, using HPLC methods described below. The percentage of intact [^68^Ga]Ga-DO3A-S01-GCG was plotted against time from administration and fitted to a mono-exponential function.

### PET procedures: Test-retest reproducibility and receptor occupancy after pre-treatment with acylated specific GCGR agonist 1-GCG

Test-retest and receptor occupancy studies were performed in NHPs (n = 5, weight 5.0 ± 1.2, range 3.8–6.5 kg) by PET/CT at the UU site, using the parameters optimised in the dose escalation study described above.

In the morning, a baseline [^68^Ga]Ga-DO3A-S01-GCG PET/CT dynamic examination over 90 minutes was performed (i.e. [^68^Ga]Ga-DO3A-S01-GCG compound corresponding to approximately 0.05 µg/kg peptide mass, as determined having negligible peptide mass in the dose escalation study).

After the baseline PET examination, the NHP was administered a study drug (i.e. phosphate buffered saline (PBS) in case of the test-retest study or 30 µg/kg 1-GCG, an acylated selective GCGR agonist, in PBS subcutaneously (Table 2). The structure 1-GCG and the model of binding to the GCGR is shown in Fig. 1B,D. The pharmacological effects of 1-GCG were described previously^7^. Two hours post study drug (at ~c_max_ of the test compound), a second 90 minutes dynamic PET examination was performed with [^68^Ga]Ga-DO3A-S01-GCG (again administered at radioactive levels corresponding to negligible mass effect to avoid significant receptor occupancy induced by the radiotracer itself), to investigate the changes in liver binding due to the 1-GCG intervention.

**Table 2:** Administration of [^68^Ga]Ga-DO3A-S01-GCG and study drug for the test-retest and receptor occupancy studies.

ID		NHP 8	NHP 9	NHP 10	NHP 11	NHP 12
	Weight	3.9	6.1	4.0	6.4	4.5
	Type	T/R	T/R	T/R	R.O.	R.O.
Scan 1	µg/kg	0.041	0.029	0.086	0.034	0.043
	MBq/kg	0.7	0.5	0.3	0.7	0.7
Study drug		1 mL PBS	1 mL PBS	1 ml PBS	30 µg/kg GCGR agonist in PBS	30 µg/kg GCGR agonist in PBS
Scan 2	µg/kg	0.033	0.034	0.069	0.042	0.051
	MBq/kg	0.5	0.5	0.2	0.6	0.9

T/R: Test-retest and R.O.: Receptor occupancy.

### Metabolic stability of [^68^Ga]Ga-DO3A-S01-GCG

Blood samples were centrifuged at 4000 rpm for 2 minutes at 4 °C (Beckman Allegra X-22R Centrifuge, Palo Alto, USA). From the plasma, 0.5 ml was taken and an equal volume of acetonitrile was added to precipitate the proteins. The mixture was centrifuged at 13200 rpm at 4 °C for 1 min (Eppendorf 5415 R centrifuge, Eppendorf AG, Hamburg, Germany). The supernatant was filtered through a 0.2 μm nylon membrane (Corning Incorporated, Corning, NY, USA) by centrifugation at 13200 rpm at 4 °C for 1 min. 500 µl of the filtered supernatant was diluted with 1500 µl H_2_O; thereafter, 30 µl of 0.01 mM unlabelled DO3A-S01-GCG was added to the mixture. The sample preparation recovery was determined by measuring the radioactivity in the plasma, filters and pellet.

HPLC analysis was performed using a binary pump system (Gilson, Middleton, USA). The sample (1.8 ml) was injected using an automated solid phase extraction controller (ASPEC Gilson) connected to a dilutor (Gilson). The separation was performed on an Xbridge Prep BEH130 C18 (peptide separation technology) 250 mm × 10 mm, 5 µm with a 10 × 10 mm C18 security guard from the same supplier. The HPLC system was operated at a flow rate of 6 ml/min. The mobile phase consisted of 0.1% TFA in MilliQ: 0.1% TFA in Acetonitrile. Gradient elution mode was used for the separation (Gradient: 0–7 min: 5–70%, 7–12 min: 70%, 12–13 min: 70–5%, 13–15 min: 5%).

A second method was developed with a lesser gradient, to determine if any metabolites were co-eluting with the DO3A-S01-GCG peak. A UV detector (Gilson) was used to detect unlabelled DO3A-S01-GCG at 220 nm. The outlet from the detector was connected to a switching valve on the arm of the ASPEC to enable automatic fraction collection. Six fractions were collected, and the radioactivity in the fractions was measured by a well-type scintillation counter. A radio detector (Radiomatic 610TR, Packard, USA) was coupled in series with the UV detector.

### Image data processing

Image processing at each site was performed according to standard optimised procedures for each respective PET scanner. At the UUH facility, the PET list mode data were reconstructed into 33 frames (12 × 10 s, 6 × 30 s, 5 × 120 s, 5 × 300 s, 5 × 600 s) using an iterative Q-Clear 200 algorithm. At the KI facility, the PET list-mode data were reconstructed into 38 frames (9 × 10 s, 2 × 15 s, 3 × 20 s, 4 × 30 s, 4 × 60 s, 4 × 180 s, 12 × 360 s) using the ordinary Poisson-3D-ordered subset expectation maximisation (OP-3D-OSEM) algorithm (10/16 iterations/subsets, including modelling of the point spread function). For SUV analysis, the frames were later resampled to the framing sequence used at the Uppsala site.

### PET data analysis

PET image analysis was performed by the PMOD 3.7 software (PMOD Technologies Ltd. Zurich, Switzerland). Regions of Interest (ROIs) were segmented on transaxial PET projections assisted by co-registered CT images if available. Tissues (liver, spleen, heart, aorta, kidney, skeletal muscle, lung, pancreas, bone and intestine) were segmented on transaxial projections and combined into Volumes of Interest (VOIs). For the liver, the portal vein and the vena cava were excluded by assistance of CT, as well as comparison of early and late image summations.

To determine an image-derived arterial input function, single pixel ROIs were placed in the aorta to avoid partial volume effects. For the PET examinations performed at KI, ROIs for the liver, spleen, heart, aorta and kidney were delineated based on the summation images of the third PET measurement from each experimental day, for which highest radioactivity was injected due to the dose escalation study design. There was no or minimal movement of the NHP during the day, as the body was fixated in a bear hugger at both sites. The dynamic PET measurements were expressed as Standardized Uptake Values (SUV) according to Eq. 1.

1 $$SUV(\frac{1}{1})=\frac{Radioactivit{y}_{tissue}(Bq)/Volum{e}_{tissue}(cc)}{Radioactivit{y}_{injected}(Bq)/Weigh{t}_{body}(g)}$$

Graphical analysis as described by Logan *et al*.^8^ was applied to the PET dynamic data from the liver (target tissue) and spleen (expected to have minimal GGCR expression) (PKIN module, PMOD 3.7 software, PMOD Technologies Ltd. Zurich, Switzerland). Volume of Distribution (V_t_) was calculated using image-derived aortic curves as input. The aortic input curve was corrected for the plasma-to-whole blood ratios obtained from blood sampling taken during each experiment. The aortic input curve was also corrected for the metabolic stability, i.e. the percentage of native [^68^Ga]Ga-DO3A-S01-GCG remaining in the blood. Goodness of fit for the model fitting was performed in the PKIN module using the standard assessments including Chi^2^, Sum-of-squares and R^2^.

For the dose escalation study, V_t_ of the liver and spleen was plotted against the administered peptide mass dose (µg/kg) to determine the peptide mass doses, which induce negligible mass effect (baseline), the background binding and the *in vivo* dissociation constant (K_d_, defined as the peptide mass dose, which induces 50% occupancy of the receptor population in the dynamic range).

### *In vitro* autoradiography of spleen

Splenic binding of [^68^Ga]Ga-DO3A-S01-GCG was previously observed in rat *in vivo*^5^. This assay was performed to elucidate if splenic binding in human and NHP *in vivo* would be expected.

Spleen tissue was collected post-mortem from the NHP and Sprague Dawley rat. Biopsies from donor human spleen were obtained from Uppsala Biobank (sample collection 827) and approved by local ethical review board (Drn 2016/465).

The splenic biopsies were processed to 10 µm frozen tissue sections by a cryotome. The sections were pre-incubated in phosphate-buffered saline (PBS) (pH 7.4, 1% Bovine Serum Albumin (BSA)) for 10 min at room temperature (RT). Then, the sections were incubated with 5 nM [^68^Ga]Ga-DO3A-S01-GCG in 150 mL PBS (pH 7.4, 1% BSA), either alone or in the presence of 10 µM GCG or 1 µm DO3A-S01-GCG precursor for 60 min at RT.

After incubation, the sections were washed three times, first with assay buffer (PBS pH 7.4 1% BSA) and then twice with PBS for 1 minute. Sections were carefully dried at 37 °C and then exposed against a digital phosphorimager plate, together with a 20 µl reference from the buffer calibrated against a gamma-counter to enable quantification to fmol bound radioligand/mm^3^. The phosphorimager plates were scanned using a Cyclone Plus Phosphor imager (Perkin Elmer) at 600 dpi, and the resulting autoradiograms analysed by ImageJ (NIH, Bethesda). The assay was repeated three times.

### Human predicted dosimetry

The predicted dosimetry of [^68^Ga]Ga-DO3A-S01-GCG in human males was estimated based on the *in vivo* biodistribution in NHPs as captured by dynamic PET examinations. The baseline PET examinations in NHP 2–7 (n=6) were averaged for the dosimetry analysis. Since the FOV of the PET scanner excluded dynamic recordings from the head and hind-limbs, we do not have complete biodistribution data for estimating dosimetry. However, the uptake in the brain in NHP is likely negligible based on observations in rat (5) as well as a whole-body PET/CT examination performed on NHP1 90–120 minutes post [^68^Ga]Ga-DO3A-S01-GCG administration.

The dosimetry calculations were performed as described previously for [^68^Ga]Ga-DO3A-VS-Cys^40^-Exendin4^4^ and [^68^Ga]Ga-DO3A-S01-GCG in rat^5^.

In brief, dynamic NHP organ uptake as measured by PET and expressed as SUV was normalised to that of human organs (using tissue weights of whole-body adult reference phantoms). The decay-corrected and normalized SUVs were back-corrected to count rates to calculate the actual radiation burden in each tissue. The tissue residence times (MBq-h/MBq) were assessed by trapezoidal approximation of the un-decay corrected human SUV biodistribution data. The tissue washout from the last time point (90 minutes) until infinity was estimated by a single mono-exponential fit.

The estimation of the absorbed dose was performed by the OLINDA/EXM 1.1 software, where the calculations were based on the adult reference male or female phantoms to obtain the absorbed dose estimate in humans (ICRP60). The organ specific doses are reported as mGy/MBq (effective dose as mSv/MBq). The amount of MBq that can be safely administered annually (MBq/year) was calculated for each organ as well as the effective dose, by dividing the limiting dose (10 mSv/year for the effective dose, 150 mGy/year for all tissues except for red marrow with 50 mGy/year) by the dose (mGy/MBq or mSv/MBq).

### Statistical considerations

Data on group level are reported as means ± SEM. Statistical analysis was performed in GraphPad Prism 6.05, and differences between groups were assessed by the students *t*-test using a significance level of p < 0.05.

### Ethical approval

All applicable international, national and/or institutional guidelines for the care and use of animals were followed.

## Results

### Radiochemistry

The radiochemical purity was 97.2 ± 1.6% (n = 28) with no unknown impurity of over 5%. Molar activity values varied depending on the age of the generator and were, on average, 95.96 ± 25.22 MBq/nmol (range 50–140 MBq/nmol) at the end of the synthesis. The reproducibility was high with non-decay corrected radiochemical yield of 59.3 ± 1.9 and success rate of 100%.

### Biodistribution in non-human primate

**Figure 2:**
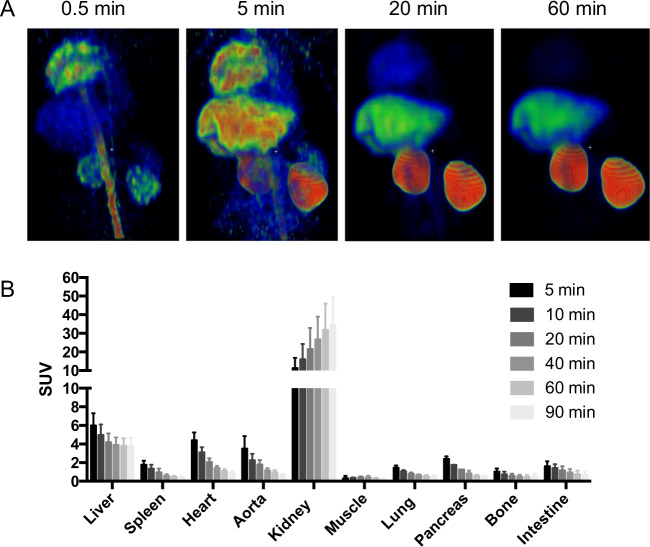
Biodistribution of [^68^Ga]Ga-DO3A-S01-GCG in NHP. Representative Maximum Intensity Projections (MIP) of biodistribution at four different time-points following [^68^Ga]Ga-DO3A-S01-GCG administration. (**A**) Quantification of tissue biodistribution by SUV measurements show that radioactivity retention occurs in the liver as well as kidney, while remaining tissues exhibit clearance of the radioactivity signal. (**B**) Bars represent averages ± SD from PET/CT examinations in n = 6 NHPs.

The biodistribution is based on the baseline scans of NHP 2–7 (n = 6). Early accumulation was seen in the vasculature and the cardiac ventricles (Fig. 2A, far left panel). A few minutes following administration, accumulation visibly occurred in liver, which has a high expression of GCGR (Fig. 2A, mid left panel). Clearance from the vasculature was apparent after 20 minutes while the liver binding was retained (Fig. 2A, mid right panel). At the late stages of the PET examinations, radioactivity retention was observed only in the liver and kidney (Fig. 2A, far right panel). The kidney accumulation was concentrated to kidney cortex. Quantification of [^68^Ga]Ga-DO3A-S01-GCG biodistribution by SUV calculation confirmed that radioactivity retention occurred in the liver and kidney, while the tracer was cleared in all other examined tissues (Fig. 2B).

### SUV analysis of dose escalation study

Table 1 describes the dose escalation study design with the administered [^68^Ga]Ga-DO3A-S01-GCG radioactivity and administered DO3A-S01-GCG peptide mass for each PET examination in each NHP.

**Figure 3:**
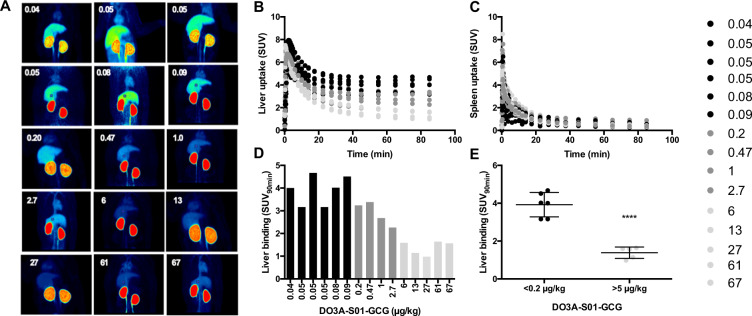
*In vivo* competition of [^68^Ga]Ga-DO3A-S01-GCG in NHP. Maximum Intensity Projections (MIPs, summed 0–90 minutes) of all PET examinations in the study, ranked after ascending DO3A-S01-GCG peptide mass dose. (**A**) SUV quantification of the dynamic uptake for all PET examinations in the liver (**B**) and spleen (**C**). SUV_90min_ as endpoint for liver binding of [^68^Ga]Ga-DO3A-S01-GCG was inversely proportional to the increase in co-injected DO3A-S01-GCG peptide mass dose. (**D**) There was a clear difference in liver binding, assessed as SUV_90min_, between the groups with the lowest (<0.2 µg/kg) and highest (≥5 µg/kg) administered peptide mass doses. ****Indicate p < 0.001.

Coronal Maximum Intensity Projections (MIPs, summed 0–90 minutes) of all PET examinations in the study visualised the strong accumulation of [^68^Ga]Ga-DO3A-S01-GCG in the liver and kidney (Fig. 3A). Increasing the co-injected DO3A-S01-GCG peptide mass progressively reduced the visual accumulation in the liver but not kidney. Temporally resolved SUV time-activity curves confirm the dose dependent decrease in [^68^Ga]Ga-DO3A-S01-GCG retention in liver (Fig. 3B). No competition effect was seen in the spleen (Fig. 3C).

SUV_90min_ was chosen as endpoint since retention differences were increasingly pronounced at later time-points. Dose-dependency was seen for the reduction in SUV_90min_ in the liver (Fig. 3D). NHPs receiving [^68^Ga]Ga-DO3A-S01-GCG solutions spiked with >5 µg/kg of co-injected peptide exhibited decreased binding (SUV_90min_ = 1.35 ± 0.29, variability 21.4%) compared to NHPs that received [^68^Ga]Ga-DO3A-S01-GCG tracer only (defined as <0.2 µg/kg co-injected peptide) (SUV_90min_ = 3.93 ± 0.67, variability 17.0%) (p < 0.0001) (Fig. 3E).

### Logan graphical analysis of dose escalation study

Metabolite stability analysis in venous plasma (n = 3) showed that the majority of radioactivity corresponded to intact [^68^Ga]Ga-DO3A-S01-GCG (95.9 ± 1.2% after 5 minutes, 89.0 ± 2.7% after 30 minutes and 80.3 ± 3.3% after 60 minutes). Based on the low observed inter-individual variability in [^68^Ga]Ga-DO3A-S01-GCG metabolic stability, a population-based model was applied to the graphical analysis in all NHPs. The plasma-to-whole blood ratios, used for correction of the image derived aortic input curve, were linear with time for all examinations and varied between 1.4 and 1.6.

**Figure 4:**
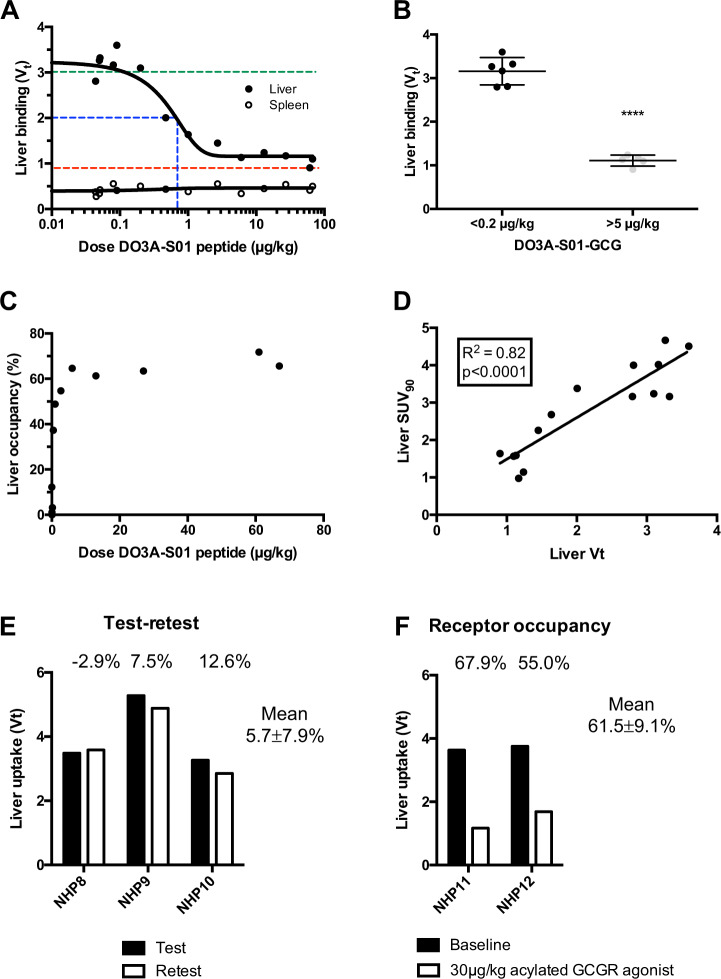
Logan graphical analysis of [^68^Ga]Ga-DO3A-S01-GCG binding. Logan derived V_t_ in the liver and spleen plotted against co-injected DO3A-S01-GCG peptide mass dose (**A**), where the green dotted line indicates <5% occupancy i.e. negligible mass effect, the blue dotted line indicates *in vivo* K_d_, the red _d_otted line indicates the background. Co-injected peptide doses >5 µg/kg strongly and consistently decreased the liver binding of [^68^Ga]Ga-DO3A-S01-GCG compared to administration of doses with negligible mass effect. (**B**) Occupancy incurred by co-injected amount of DO3A-S01-GCG peptide mass dose. (**C**) Correlation between Logan derived V_t_ and SUV_90min_ in the liver (**D**). ****Indicates p < 0.0001. Test-retest variability of [^68^Ga]Ga-DO3A-S01-GCG binding in the liver over the course of an experimental day. (**E**) Effect on [^68^Ga]Ga-DO3A-S01-GCG binding in the liver by pre-treatment with 30 µg/kg of the acylated GCGR agonist 1-GCG (**F**).

V_t_ as estimated by Logan graphical analysis using metabolite corrected image derived arterial plasma input showed strong dose dependency (Fig. 4A). Negligible mass effect (defined as <5% occupancy incurred from baseline 3.2 µg/kg, green dotted line) was achieved at DO3A-S01-GCG doses below approximately 0.2 µg/kg. The *in vivo* K_d_ in the liver, i.e. 50% incurred occupancy from baseline in the dynamic range (grey shaded area) above the background (red dotted line), was estimated to 0.7 µg/kg. Peptide mass dose escalation of co-injected DO3A-S01-GCG progressively decreased the V_t_ parameter to levels where only minor further additional blocking effect was seen (background level V_t_ = 0.9). V_t_ in the spleen was low for all PET examinations and unaffected by competition, as required from a suitable reference tissue. Group analysis of co-injected peptide doses >5 µg/kg (V_t_ = 1.11 ± 0.13, variability 11.5%) decreased the binding compared to administration of doses with negligible mass effect <0.2 µg/kg (V_t_ = 3.16 ± 0.31, variability 9.9%) (p < 0.0001) (Fig. 4B). The occupancy incurred in the liver by co-injection of the different doses of DO3A-S01-GCG peptide, calculated from the decrease in [^68^Ga]Ga-DO3A-S01-GCG binding from baseline V_t_ = 3.2, reached maximally 72% (Fig. 4C). Liver SUV_90min_ as simplified endpoint correlated linearly with the Logan derived V_t_ (Fig. 4D).

### Test-retest reproducibility and receptor occupancy after pre-treatment with an acylated specific GCGR agonist

Test-retest reproducibility of [^68^Ga]Ga-DO3A-S01-GCG binding in the liver, as assessed over the course of the same day by pre-treatment with PBS before follow-up scanning, was 5.7 ± 7.9% (n = 3) (Fig. 4E). Pre-treatment with an acylated specific GCGR agonist, 1-GCG, at a dose of 30 µg/kg, on the other hand, resulted in a distinct decrease in the liver binding of 61.5 ± 9.1% (n = 2) (Fig. 4F).

### *In vitro* autoradiography of [^68^Ga]Ga-DO3A-S01-GCG in the spleen

Previously, we reported selective *in vivo* binding of [^68^Ga]Ga-DO3A-S01-GCG in rat spleen in biodistribution studies^5^. In the NHP biodistribution study described above, no splenic uptake, or effect by competition, was seen. *In vitro* autoradiography studies of splenic sections were therefore performed to understand if binding is to be expected in the human spleen.

**Figure 5:**
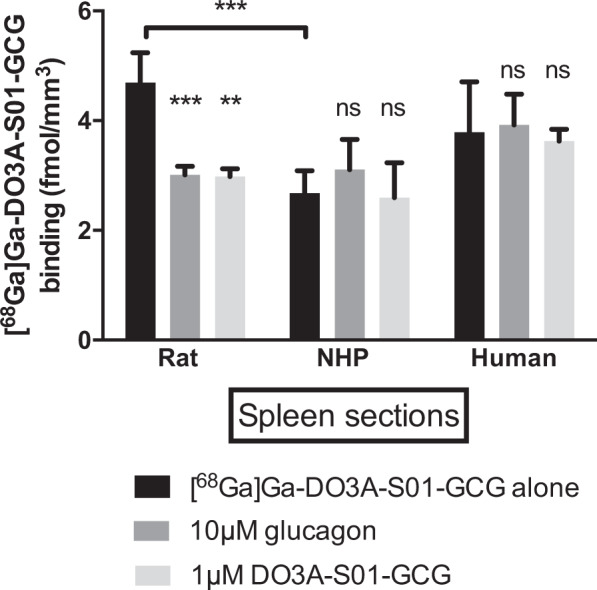
*In vitro* autoradiography of [^68^Ga]Ga-DO3A-S01-GCG binding in frozen sections of spleen. [^68^Ga]Ga-DO3A-S01-GCG binding in rat could be inhibited by both endogenous glucagon and unlabelled DO3A-S01-GCG in excess. No strong binding was seen in splenic sections from NHP or human, and co-incubation with endogenous glucagon or unlabelled DO3A-S01-GCG did not affect the binding. Bars represent averages ± SD from n = 3 separate experiments.

[^68^Ga]Ga-DO3A-S01-GCG binding to sections of rat spleen was strong, and could be blocked to a similar degree by co-incubation with both endogenous glucagon and unlabelled DO3A-S01-GCG precursor peptide in excess (Fig. 5).

The binding of [^68^Ga]Ga-DO3A-S01-GCG to splenic sections from NHP and human was lower than in rat. Importantly, the binding was not affected by co-incubation with either endogenous glucagon or unlabelled DO3A-S01-GCG precursor peptide.

### Human predicted dosimetry

**Figure 6:**
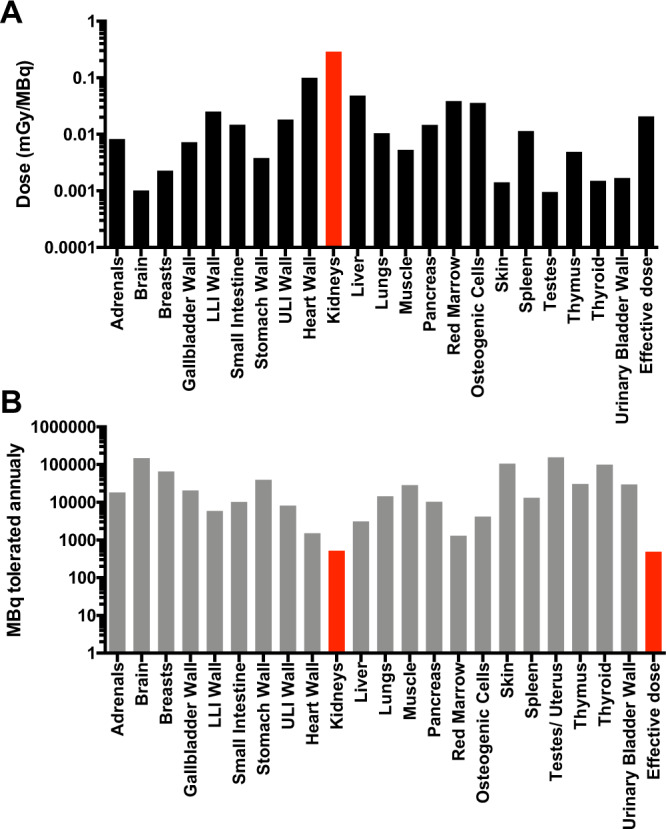
Human dosimetry of [^68^Ga]Ga-DO3A-S01-GCG as predicted from NHP biodistribution. The human predicted tissue specific absorbed dose and effective dose (in mSV/MBq) of [^68^Ga]Ga-DO3A-S01-GCG. (**A**) The maximal possible annual radioactive dosing of [^68^Ga]Ga-DO3A-S01-GCG, based on the human predicted absorbed dose and the annual radiation safety limits. (**B**) Red bars indicate the critical organ. Logarithmic scale (Y-axis) is used for both panels.

The predicted human dosing per MBq administered [^68^Ga]Ga-DO3A-S01-GCG as extrapolated from NHP biodistribution is presented in Fig. 6A. Kidneys received the highest absorbed dose (0.29 mGy/MBq, red bar), followed by the heart wall (myocardium) (0.10 mGy/MBq) and liver (0.049 mG/MBq). Red marrow received 0.039 mGy/MBq. The effective dose of [^68^Ga]Ga-DO3A-S01-GCG was 20.6µSv/MBq.

Based on the absorbed dose and the annual limit for absorbed dose for each tissue, there is a limit of 485 MBq [^68^Ga]Ga-DO3A-S01-GCG annually, where the effective dose is limiting (Fig. 6B). The critical organ is the kidney, which would allow for an annual administration of 519 MBq [^68^Ga]Ga-DO3A-S01-GCG. The absorbed dose for [68Ga]Ga-DO3A S01-GCG in red marrow of NHP predicts an annual exposure limit for humans in excess of 1000 MBq.

## Discussion

We present evidence of a first-in-class radioligand, capable of sensing and quantifying drug activity at the GCGR *in vivo* in NHP by PET. [^68^Ga]Ga-DO3A-S01-GCG was previously evaluated *in vitro* and *in vivo* in rat, wherein nanomolar affinity and strong specificity for the GCGR were demonstrated^5^. [^68^Ga]Ga-DO3A-S01-GCG bound in rat liver *in vivo* in a receptor mediated manner. Here, we demonstrate that the results also translate into a large animal model with high fidelity for human physiology.

When using a radioligand to study a receptor system in a relatively unperturbed physiological baseline state (ignoring possible effects of anesthesia), it is imperative that the administered radioligand mass itself does not illicit a mass effect i.e. does not carry a molar abundance that significantly affects the receptor system. Negligible mass effect of a radioligand is usually defined as exerting less than 5% receptor occupancy.

The dose escalation study was primarily intended to reveal the upper limit of mass injection not evoking significant receptor occupancy. In addition, the procedure allowed us to evaluate the proportionality between administered competing DO3A-S01-GCG peptide dose and the magnitude of binding of [^68^Ga]Ga-DO3A-S01-GCG in liver. The increase in radioactivity for each subsequent PET examination means that residual remaining radioactivity in the NHP from previous examinations during the same experimental day will be negligible. The increase in peptide mass (dose escalation) for each subsequent PET/CT examination means that the previously administered peptide mass during the experimental day will be negligible or very low in comparison.

NHPs were dosed with [^68^Ga]Ga-DO3A-S01-GCG corresponding from 0.04 µg/kg up to 67 µg/kg DO3A-S01-GCG peptide mass. Due to the limit imposed by molar activity, it was not possible to administer a mass dose less than 0.04 µg/kg while still retaining sufficient injected radioactive signal. The binding in the liver, using either V_t_ or the simpler SUV_90min_, showed clear dose dependency. The *in vivo* K_d_ of [^68^Ga]Ga-DO3A-S01-GCG in the dynamic range in the liver was 0.7 µg/kg, which is in line with the strong potency for NHP GCGR demonstrated *in vitro*^5^. The maximal binding plateaued at approximately V_t_ = 3.2. DO3A-S01-GCG peptide mass doses <0.2 µg/kg were estimated to induce negligible mass effect. Assuming that the NHP data are predictive of the human situation, this implies that [^68^Ga]Ga-DO3A-S01-GCG should be administered in doses <0.2 µg/kg in clinical trials for accurate measurement of GCGR occupancy in the liver.

Only minor additional competition effect was seen for administered doses in excess of 6 µg/kg. [^68^Ga]Ga-DO3A-S01-GCG background binding in the liver was approximately V_t_ = 0.9, which indicated a retention process that differs from, for example, the spleen where the background binding was approximately V_t_ = 0.4. This residual background signal in the liver was the reason for not achieving more than 72% occupancy at the highest competing doses. Xenobiotics are often metabolised in the liver, which may partly account for the slightly elevated hepatic background. Pre-treatment with a high dose (30 µg/kg) of acylated selective GCGR agonist, 1-GCG, resulted in a similar magnitude of occupancy, up to almost 70%. In a previous study, 1-GCG was tested at 30 µg/kg with repeated s.c. dosing in obese diabetic NHP over 4 weeks, where it led to elevated glucose excursion and increased diabetogenic risk whereas, when dosed on top of a selective GLP-1R agonist, it led to increased body weight loss but somewhat impaired glucose control compared to treatment with the selective GLP-1R alone^7^.

The Logan graphical model provided acceptable goodness of fit for the PET data from all scans regardless magnitude of liver binding, including the baseline scans (Chi^2^ = 4.9, R^2^ = 0.987), the full precursor blockade scans (Chi^2^ = 2.1, R^2^ = 0.999), the 1-GCG pre-treatment scans (Chi^2^ = 4.8, R^2^ = 0.996) and the re-test scans (Chi^2^ = 2.0, R^2^ = 0.996).

There was a linear correlation between V_t_ and SUV_90min_, indicating that SUV_90min_ can serve as a simplified estimate for GCGR occupancy. However, SUV_90min_ exhibited larger variability on a group level (SUV_90min_ variability = 17.0%) than V_t_ (V_t_ variability = 9.9%), which uses a larger portion of the dynamic examination for its calculation. By using the image-derived arterial plasma, corrected for metabolic stability, as input for advanced image analysis, it was possible to correct for individual differences in blood flow, radioligand metabolic stability, clearance from the vasculature, etc. These corrections to the tissue radioactive signal are designed to increase precision of the measurement, which is particularly important when considering quantification of occupancy on a receptor. V_t_ is therefore recommended for highest possible precision in measurement, despite the more demanding analysis process. The current study was performed on two different PET scanners with different spatial resolution. However, partial volume effects are not expected given the large size of the liver compared with individual pixels. For the delineation of aorta, only single pixels deemed within lumen were included for the image derived input signal, to minimize any partial volume effects regardless of scanner.

No specific [^68^Ga]Ga-DO3A-S01-GCG binding was observed in the NHP spleen as opposed to in rat spleen^5^. Spleen was therefore proposed as a reference tissue due to its proximity to the liver (i.e. fitting in the same PET FOV), the relative ease of delineation and importantly the lack of specific binding of [^68^Ga]Ga-DO3A-S01-GCG in either NHP or human splenic sections as demonstrated by autoradiography. Specific bone marrow uptake was also seen in rat, but not in NHP. GCGR expression on the protein level has not been reported in either human spleen or bone marrow in the Human Protein Atlas^9^. Thus, neither spleen nor bone marrow binding of [^68^Ga]Ga-DO3A-S01-GCG is expected in human.

The kidney uptake was not affected by the dose escalation, strongly indicating that it is not mediated by GCGR uptake. Radiolabelled small peptides are often excreted in urine and reabsorbed by the renal tubules in the kidney cortex^10^. Generally, residualising radiometal-based peptide PET tracers (such as Gallium-68 based ones) are trapped in the renal tubules following the reabsorption, and it is highly likely that the kidney uptake of [^68^Ga]Ga-DO3A-S01-GCG as seen here is mediated by this process.

Binding of [^68^Ga]Ga-DO3A-S01-GCG in GLP-1R rich pancreas was low (SUV_90min_ < 1), thus, further reinforcing the lack of cross-binding. As comparison, the GLP-1R selective PET radioligand [^68^Ga]Ga-DO3A-VS-Cys40-Exendin-4 exhibits strong pancreatic binding (SUV_90min_ > 6), while displaying negligible binding in the liver^4^.

The manual preparation of [^68^Ga]Ga-DO3A-S01-GCG yielded a molar activity (i.e. the efficiency of the radiolabelling, or the amount of MBq radioactivity incorporated per nanomole of the peptide) of, on average, almost 100 MBq/nmol at the end of the synthesis. The preparation method demonstrated high reproducibility with 100% success rate, and the variation in molar activity values was solely due to the age of the generator and thus total radioactivity amount available. Even the minimal value of 50 MBq/nmol allowed for low administered peptide mass while providing sufficient radioactivity for statistically relevant counts and high-quality PET images. However, a clinical study would require good manufacturing practice compliant automation of [^68^Ga]Ga-DO3A-S01-GCG production, which may result in lower achievable SRA. Assuming a human weight of 75 kg, administered radioactivity dose of 1 MBq/kg, and SRA of 30 MBq/nmol, preliminary estimation showed that the administered peptide dose would correspond to 0.17 µg/kg. Such low peptide dose would assure adequate assessment of GCGR receptor occupancy in humans.

The human predicted dosimetry of [^68^Ga]Ga-DO3A-S01-GCG extrapolated from NHP exhibited some discrepancies from the dosimetry calculation based on rat biodistribution published previously^5^. The most pronounced discrepancy was the higher absorbed dose to the kidneys in rat compared to NHP. In contrast, most other organs showed lower absorbed doses based on rat data. This is possibly due to a more rapid metabolism in wake rats compared to anaesthetised NHPs, where faster clearance from the blood stream leads to faster reabsorption and trapping in renal tubules – i.e. increased renal exposure. The radioactivity in the blood as well as in most peripheral tissues is consequently higher in NHP than in rat due to comparatively lower excretion in the larger animal model. The dosimetry calculation takes cross-firing (i.e. the radioactive dosing to an organ from a neighbouring tissue) from the blood stream into account for tissue-absorbed dose. This is especially noticeable in the heart wall based on NHP data, which receives the majority of its dose from passing blood volume.

Human predicted dosimetry as extrapolated from NHP allows for annual administration of IV 485 MBq [^68^Ga]Ga-DO3A-S01-GCG based on the effective dose and >500 MBq based on the critical organ kidney, meaning that GCGR occupancy can be measured repeatedly in humans without exceeding radiation safety limits. Assuming a radioactivity dose of 1 MBq/kg [^68^Ga]Ga-DO3A-S01-GCG for human studies (75 MBq in a 75 kg individual), up to 6 PET examinations of GCGR occupancy could potentially be performed annually. [^68^Ga]Ga-DO3A-S01-GCG is therefore suitable for interventional studies, where repeated examinations are required.

In the context of dual GLP-1/GCG receptor agonist development, [^68^Ga]Ga-DO3A-S01-GCG as a PET radioligand provides the means to demonstrate target engagement at the GCGR early in the drug development process. GLP-1R radioligand [^68^Ga]Ga-DO3A-VS-Cys40-Exendin-4 has similarly been employed for showing target engagement of intravenously administered Exendin-4 in the pancreas^11^.

These two complementing tools potentially give access to direct quantification of drug activity at both the GCGR and GLP-1R in humans, and are expected to assist in unravelling the contribution of respective hormone receptors to the beneficial physiological effects seen during dual GLP-1/GCG receptor agonist treatment.

In summary, first-in-class GCGR PET radioligand [^68^Ga]Ga-DO3A-S01-GCG bound specifically in the NHP liver and could be competed away dose-dependently. Occupancy at the GCGR was quantifiable and dose dependent. Administration of [^68^Ga]Ga-DO3A-S01-GCG at peptide doses <0.2 µg/kg incurred negligible mass effect and constituted GCGR imaging at basal undisturbed physiological conditions. Test-retest reproducibility was excellent, and proof-of-concept for *in vivo* assessment of occupancy at the GCGR in the liver was demonstrated with an acylated glucagon agonist. Repeated examinations can be performed in humans without exceeding radiation safety limits. [^68^Ga]Ga-DO3A-S01-GCG has the potential to become an important tool in dual GLP-1/GCG receptor agonist drug candidate selection and optimisation in clinical trials.
